# Accidental finding of COVID‐associated mucormycosis (CAM) in a patient presenting as toothache: A case report and review of literature

**DOI:** 10.1002/ccr3.7292

**Published:** 2023-05-04

**Authors:** Protyusha GB, Kavitha B, B. Sivapathasundharam, P. Manodh, A. Thirumal Raj, Snehashish Ghosh, Safal Dhungel

**Affiliations:** ^1^ Department of Oral Pathology and Microbiology Meenakshi Ammal Dental College and Hospital Chennai India; ^2^ Priyadarshini Dental College and Hospital Chennai India; ^3^ Department of Oral and Maxillofacial Surgery Meenakshi Ammal Dental College and Hospital Chennai India; ^4^ Department of Oral Pathology and Microbiology Sri Venkateshwara Dental College and Hospital Chennai India; ^5^ Department of Oral Pathology College of Medical Sciences Bharatpur Nepal; ^6^ Department of Oral and Maxillofacial Surgery College of Medical Sciences Bharatpur Nepal

**Keywords:** COVID‐19, fungal, mucormycosis, pandemic, rhinocerebral

## Abstract

Mucormycosis is an angio‐invasive rapidly progressing fungal infection, usually reported in immunocompromised individuals. We present a case of COVID‐associated mucormycosis in a patient with a presenting symptom of toothache in the maxilla with a possible mild case of COVID‐19.

**Abstract:**

Coronavirus‐associated mucormycosis (CAM) had reached epidemic status, especially during the second wave of COVID‐19. It was especially prevalent in India with a large mortality rate. Mucormycosis, particularly the rhinocerebral type is seen to be greatly associated with COVID‐19, especially in patients with altered immunity. Uncontrolled diabetes, chronic kidney disease, immunocompromised patients, malignant hematological diseases, etc. are the major risk factors of CAM, precipitated by the injudicious use of corticosteroids for the treatment of COVID‐19. CAM may often present in the maxillofacial region which warrants that dental clinicians be aware of the clinical presentation, diagnostic guidelines, and appropriate management measures for the disease. This report is one such case of CAM involving the posterior maxilla in a middle‐aged individual with mild COVID‐19 symptoms.

## 1 INTRODUCTION

The coronavirus 2019 (COVID‐19) pandemic continues to be a significant health issue worldwide. Since its inception, this unique disease has displayed several twists and turns in its pathophysiology, diagnostic criteria, management, sequelae, and complications.^1^ The virus has undergone several mutations and has recurred in waves all around the globe. The second wave saw the emergence of the deadly “Delta” variant of the SARS‐CoV‐2 virus with higher mortality rates while the third wave was dominated by the lesser virulent Omicron variant. Mucormycosis in COVID‐19 patients had shown prevalence, especially during the second wave of the pandemic, and caused a stir among people worldwide and India in particular.^2^, ^3^, ^4^

COVID‐associated mucormycosis (CAM) usually evolves rapidly in immunocompromised or debilitated patients with conditions such as uncontrolled diabetes, patients on chemotherapy or corticosteroids, chronic kidney disease, malignant hematological diseases, lymphopenia, and solid organ transplantation.^5^, ^6^, ^7^, ^8^ Mucormycosis has a global prevalence of 0.005–1.7 per million population, with India reporting nearly 80 times higher cases (0.14 per 1000) according to the statistics in 2019–2020.^9^ A recent study has also designated mucormycosis as a notifiable disease in India with more than 28, 252 cases reported across the country.^8^ The association between mucormycosis and diabetes mellitus is well established with an overall mortality rate of 46%.^9^

The recent unprecedented surge in COVID‐associated mucormycosis (CAM) cases in India especially during the second wave, has garnered widespread attention due to its serious clinical manifestations and its association with diabetes and the treatment strategy of COVID‐19. Therefore it is of pivotal importance the clinicians be aware of the occurrence of CAM, as early diagnosis with effective management is the only way to reduce the morbidity and mortality associated with this disease. The present case is one such and is therefore reported.

## 2 CASE REPORT

**FIGURE 1:**
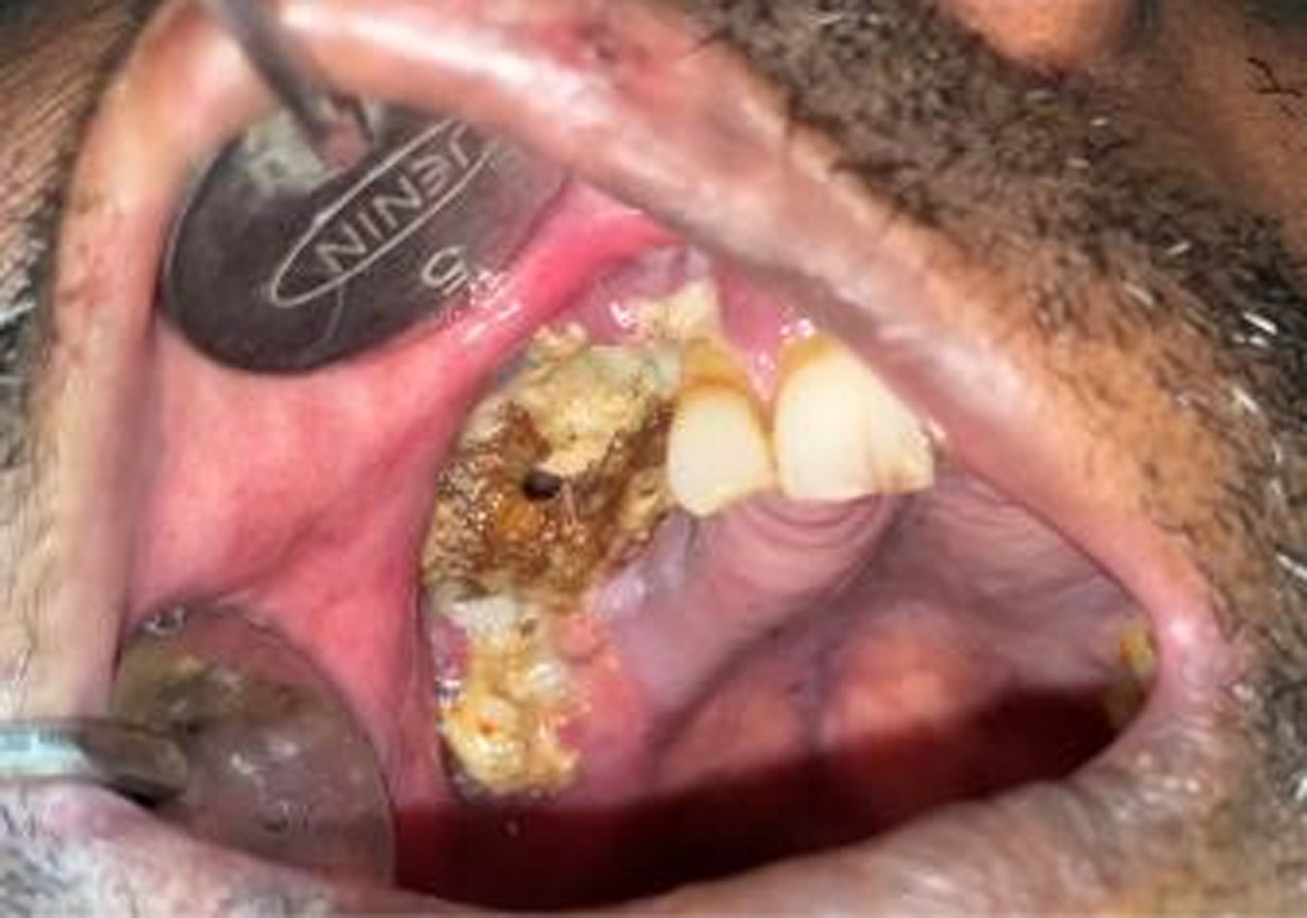
Extensive areas of necrosed bone in right upper alveolar region.

**FIGURE 2:**
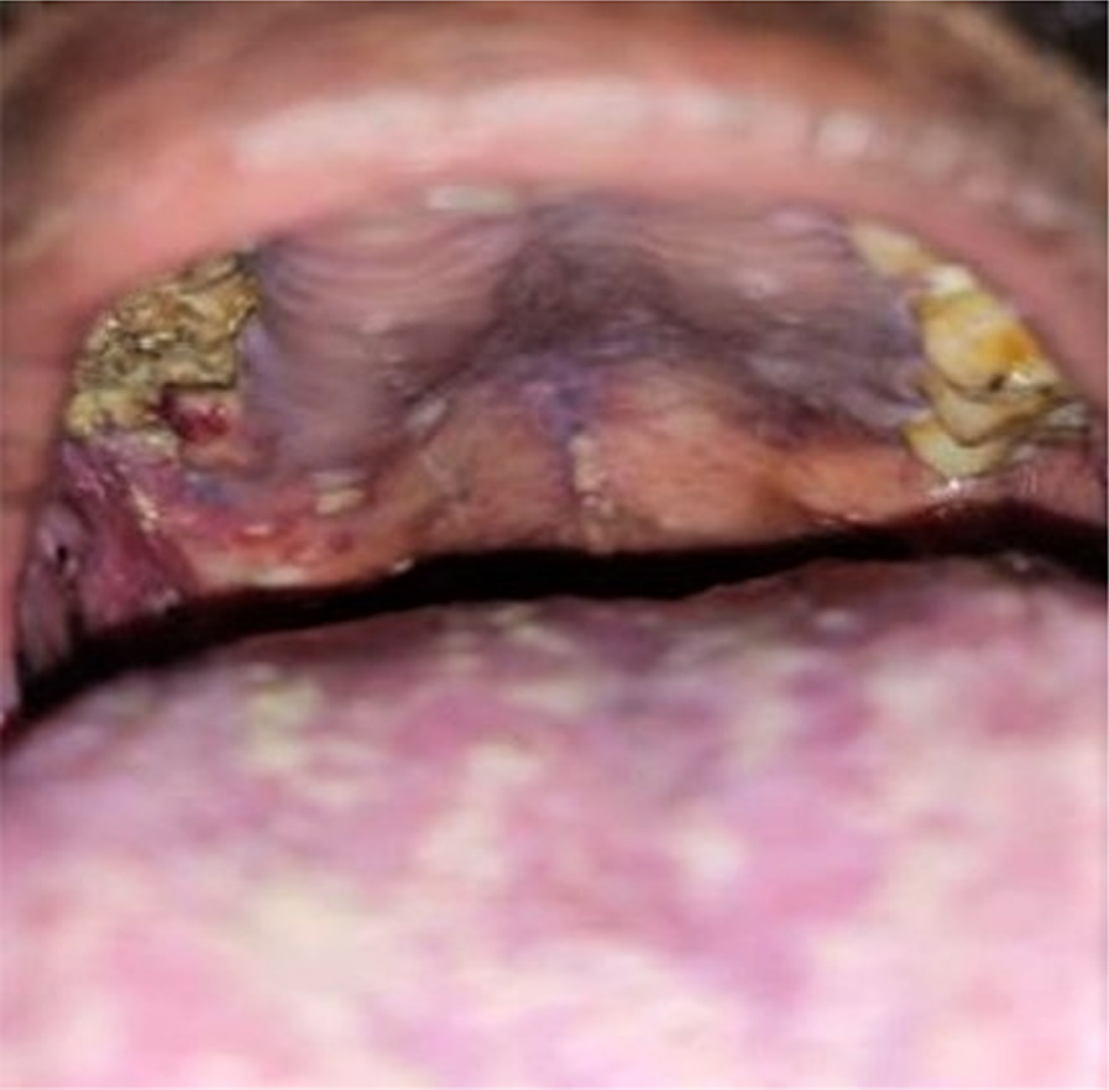
Multiple pseudomembranous, scrapable areas seen on the tongue and palate.

**FIGURE 3:**
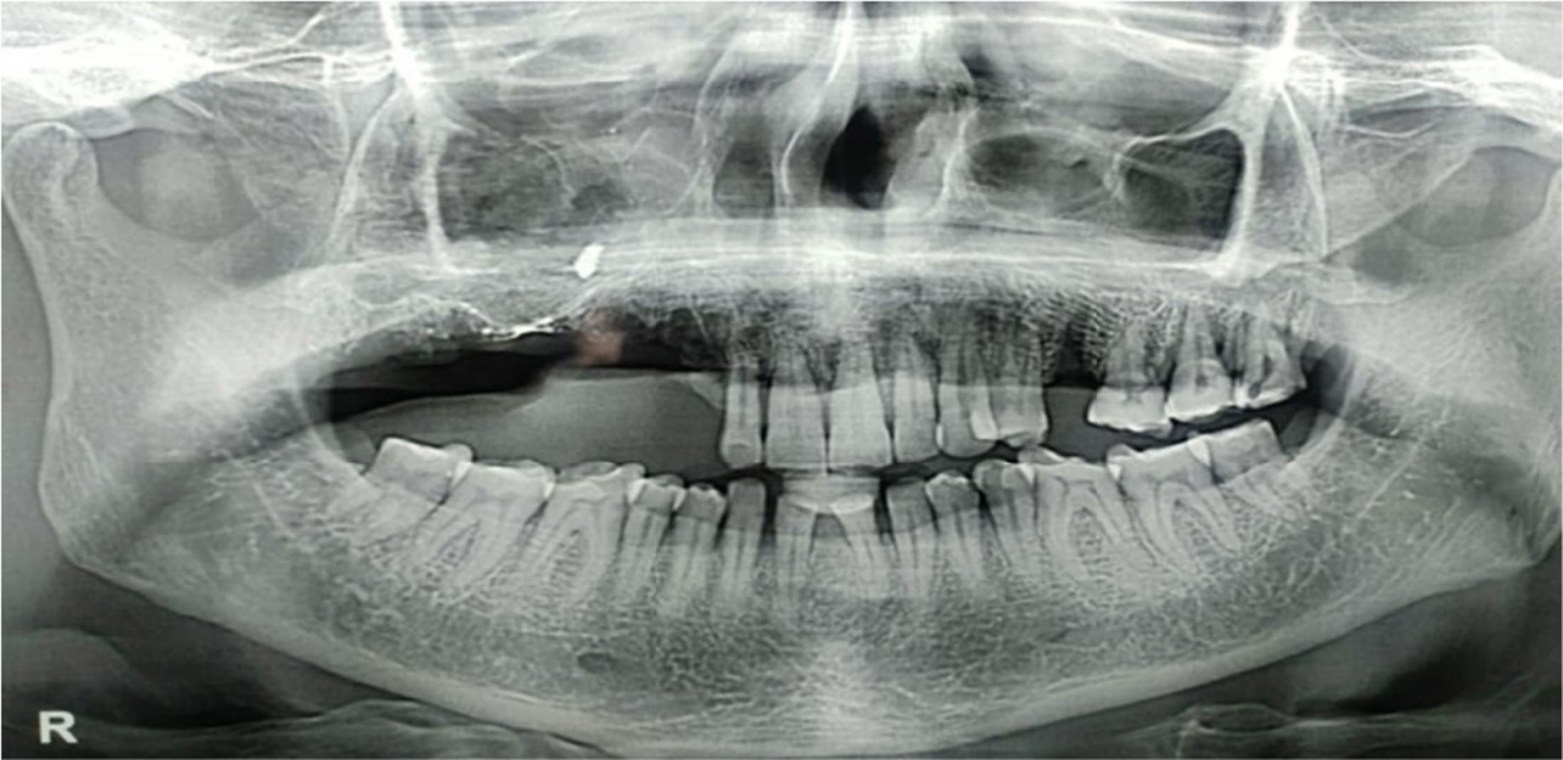
Orthopantomagram (OPG) showing significant erosion of bone with loss of teeth in the right maxilla.

**FIGURE 4:**
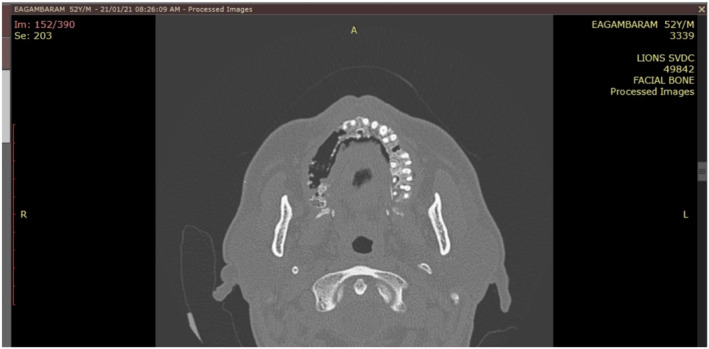
Computed tomography of maxilla (axial section) showing extensive destruction of alveolar process.

**FIGURE 5:**
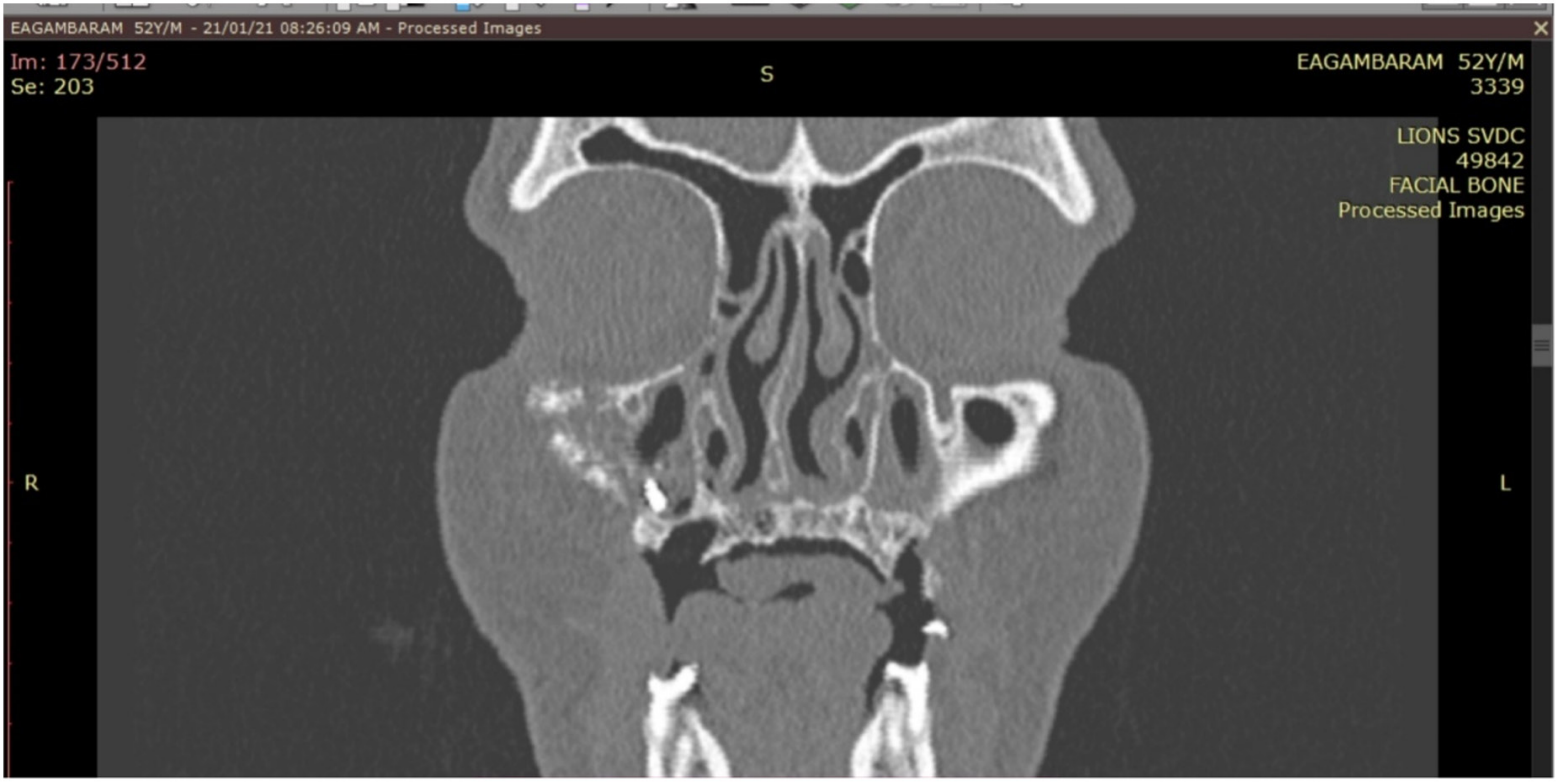
Computed tomography of paranasal sinuses (coronal section) showing lytic lesions in bilateral maxillary sinus with extension into floor of orbit.

**FIGURE 6:**
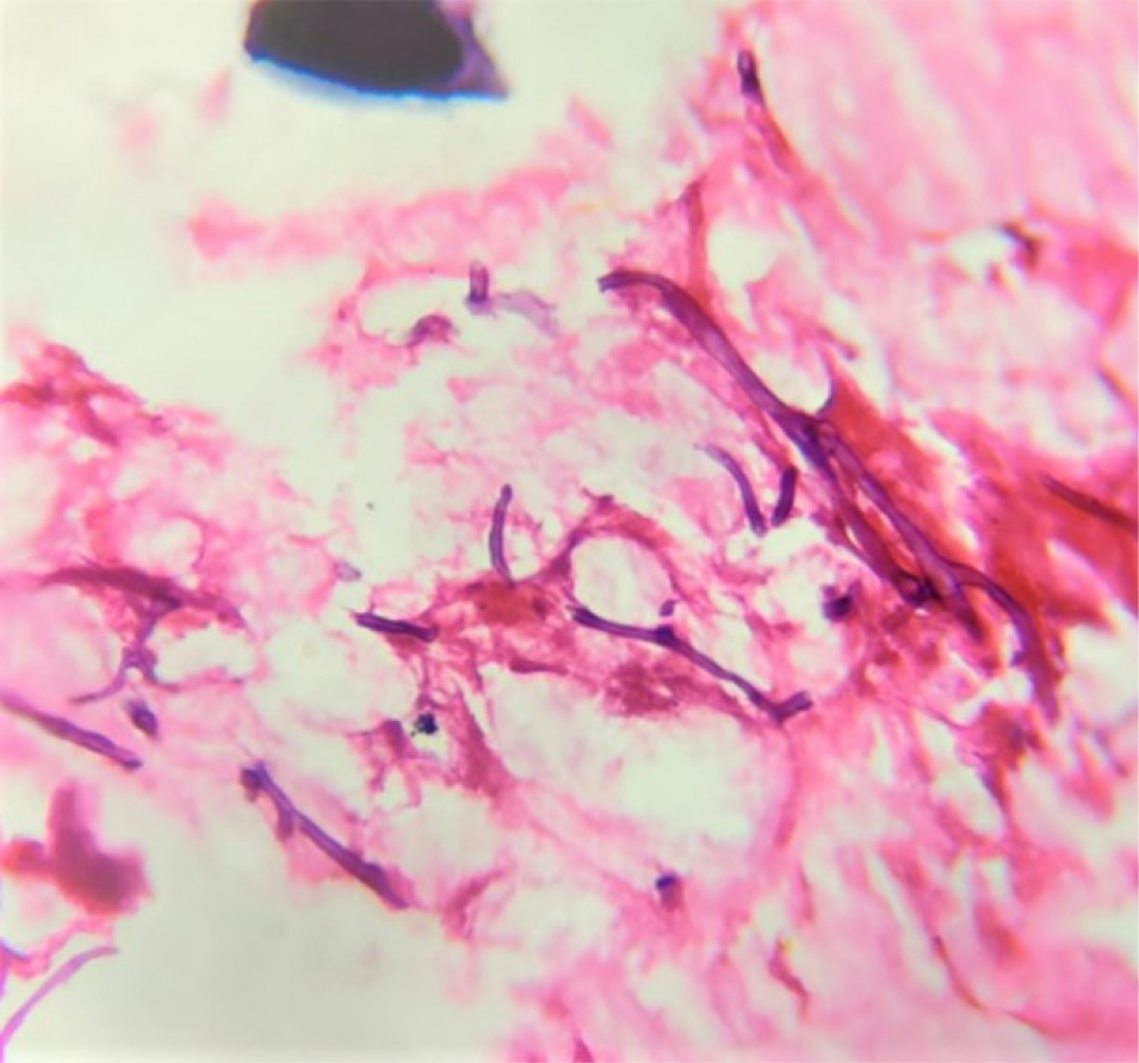
Incisional biopsy showing broad, ribbon shaped, branched aseptate fungal hyphae.

A 52‐year‐old male patient reported to the outpatient department of a dental college in southern India with the chief complaint of pain in the upper right back tooth region for the past 3 months. The visit of the patient was during the month of February 2021 which coincided with the rising COVID cases just before the delta wave in India. The history of presenting illness of the patient revealed spontaneous loosening and falling of multiple teeth in the past month with watery discharge from the nose while drinking water. The patient was a known diabetic and hypertensive with a history of fever 1 month back. He had visited a dentist earlier for the same complaint and had undergone an extraction of his mobile teeth. Furthermore, the patient had a paan (betel leaf) and tobacco‐chewing habit for the past 15 years with a history of pouching in his right upper buccal vestibule. Extraoral examination showed mild asymmetry on the left side of the face with no tenderness on palpation. The right side of the face was associated with paresthesia. Intraoral examination revealed extensive areas of necrosed bone in the upper right alveolar region extending from 13 regions to 18 regions with pus discharge (Figure 1). Oroantral communication with the watery discharge was evident with multiple pseudomembranous, scrapable areas in the buccal mucosa, tongue, and soft palate (Figure 2). The patient's HbA1c level was found to be 9.7%. OPG revealed significant erosion of bone with loss of teeth in the right maxilla (Figure 3). CT showed bone necrosis in relation to the bilateral palate, maxillary alveolus, and right zygomatic body (Figures 4 and 5). A provisional diagnosis of fungal osteomyelitis of the maxilla was made with actinomycosis and aspergillosis as the differential diagnoses. An incisional biopsy was performed. Hematoxylin and Eosin stained section showed the presence of broad ribbon‐shaped obtuse angle branched aseptate fungal hyphae, suggesting a diagnosis of mucormycosis (Figure 6). In the meantime, the patient was screened for COVID‐19 for which he tested negative. However, the SARS‐CoV‐2 antibody test showed a positive test result for the IgM antibody. The patient was started on Tab. Posaconazole 300 mg/day. Surgically, left subtotal maxillectomy with zygomatic extension and right total maxillectomy were performed and the excised mixed tissue specimens were sent for histopathological analysis.

**FIGURE 7:**
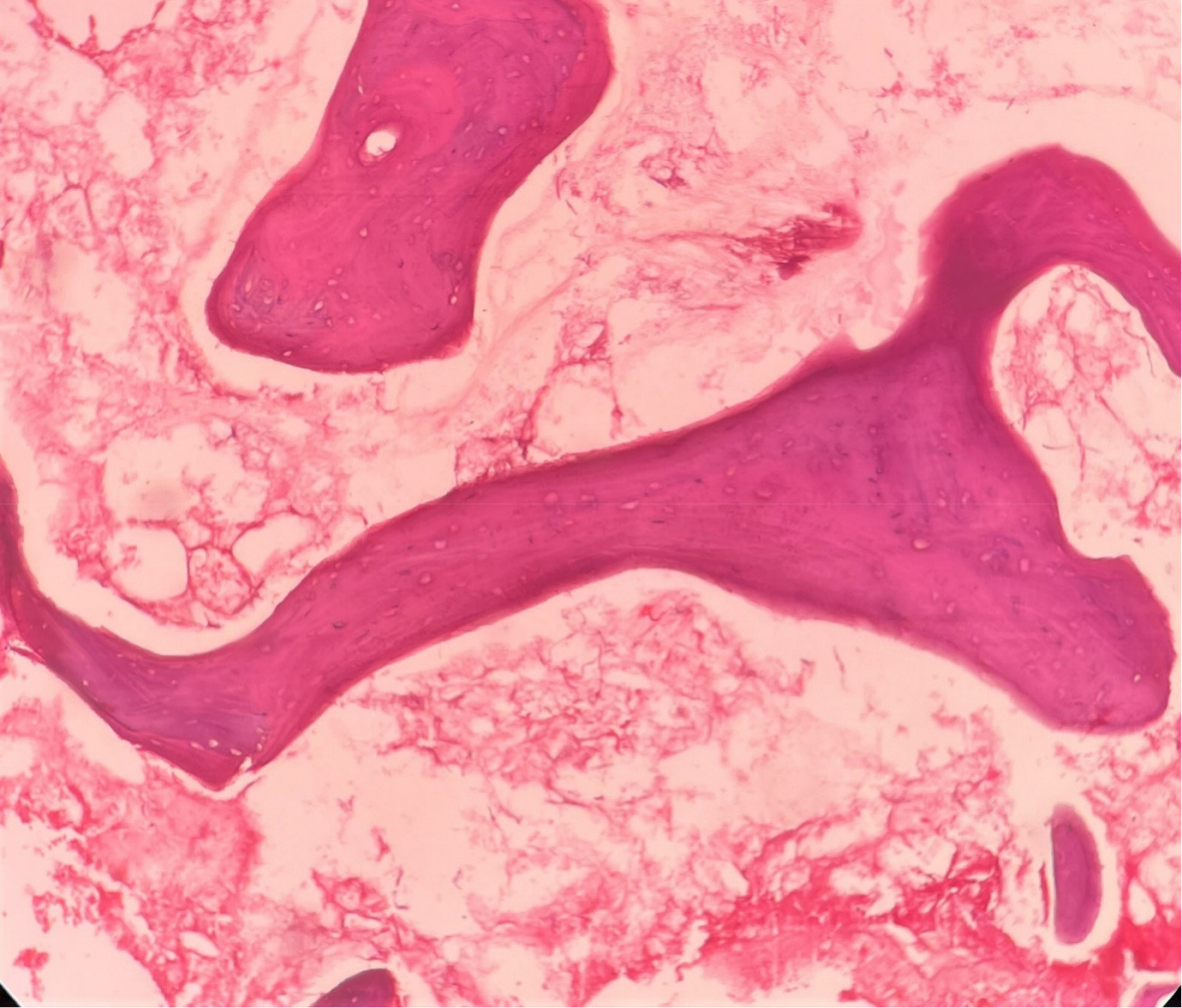
Bony trabeculae with empty osteocytic lacunae, necrotic bone marrow, and hemorrhagic areas.

**FIGURE 8:**
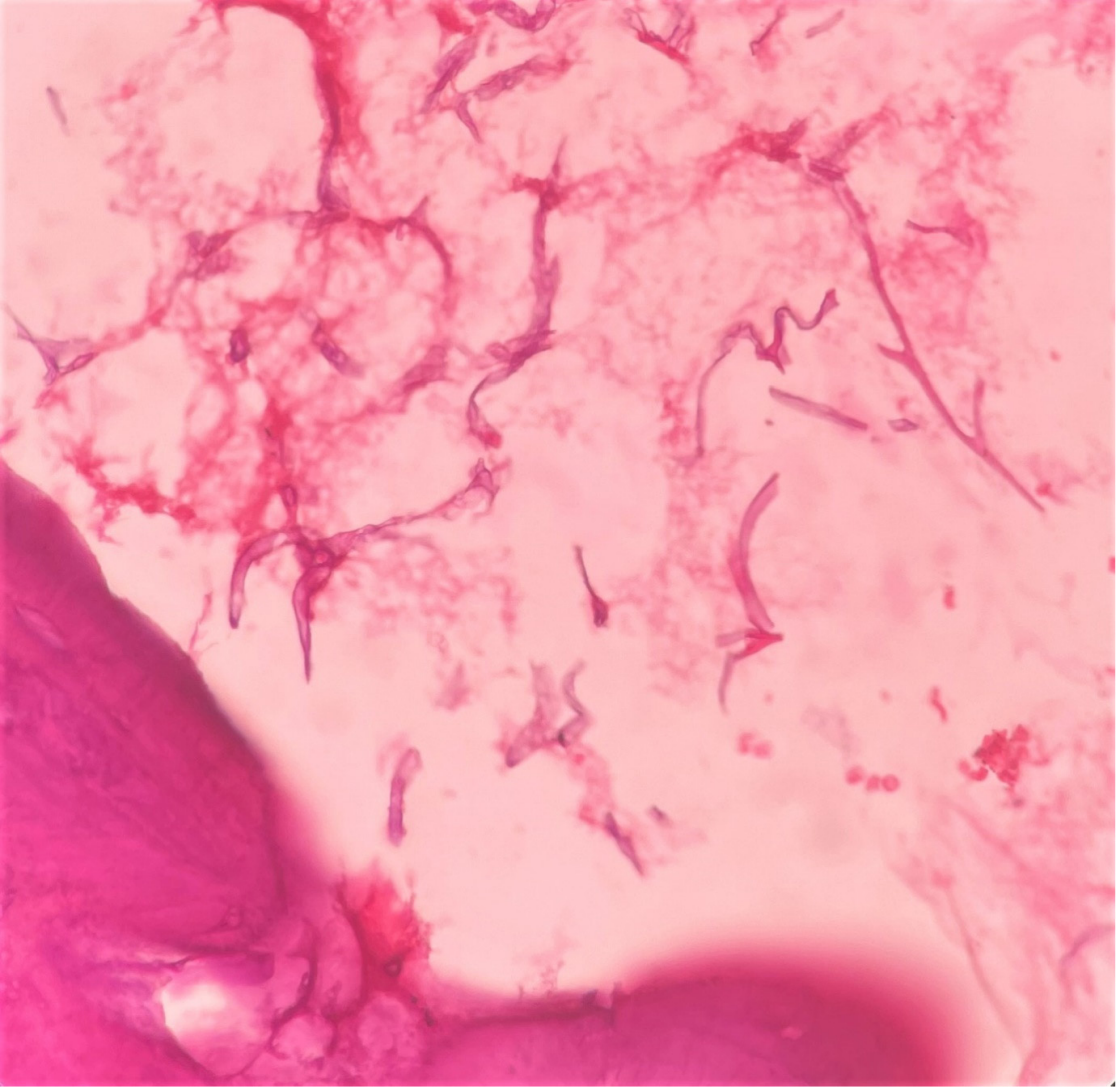
Excisional biopsy showing numerous broad, ribbon shaped, branched aseptate fungal hyphae.

**FIGURE 9:**
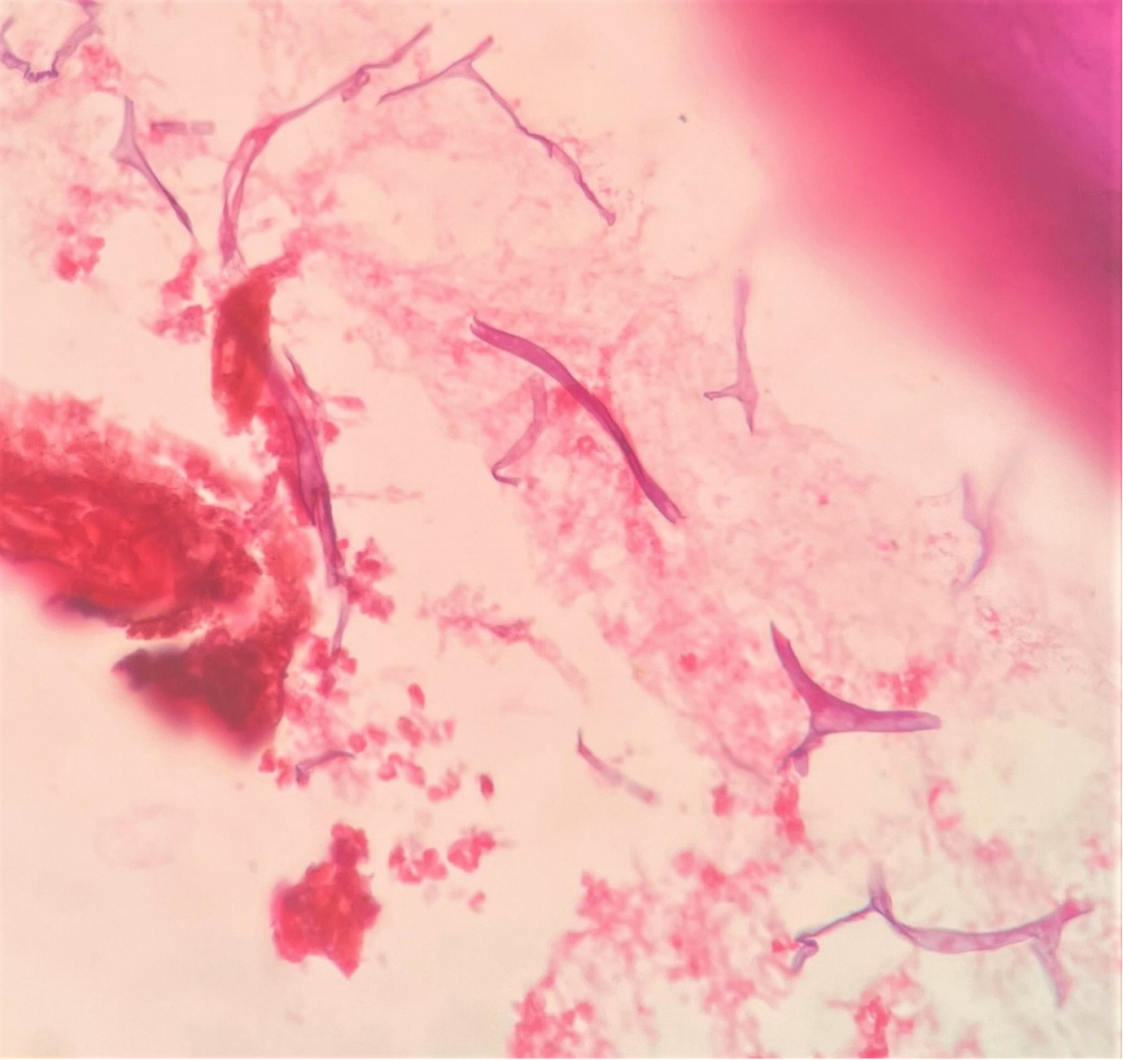
Aseptate fungal hyphae branched at obtuse angle.

On histopathological examination, decalcified sections showed numerous fragmented bony trabeculae with empty osteocytic lacunae, necrotic bone marrow, and areas of hemorrhage (Figure 7). Also, numerous broad ribbon‐shaped branched aseptate fungal hyphae are seen (Figures 8 and 9). The nasal floor and sinus mucosa showed pseudostratified ciliated columnar epithelium with underlying fibrous connective tissue exhibiting lymphoplasmacytic infiltration. Deeper layers show seromucous glands.

**FIGURE 10:**
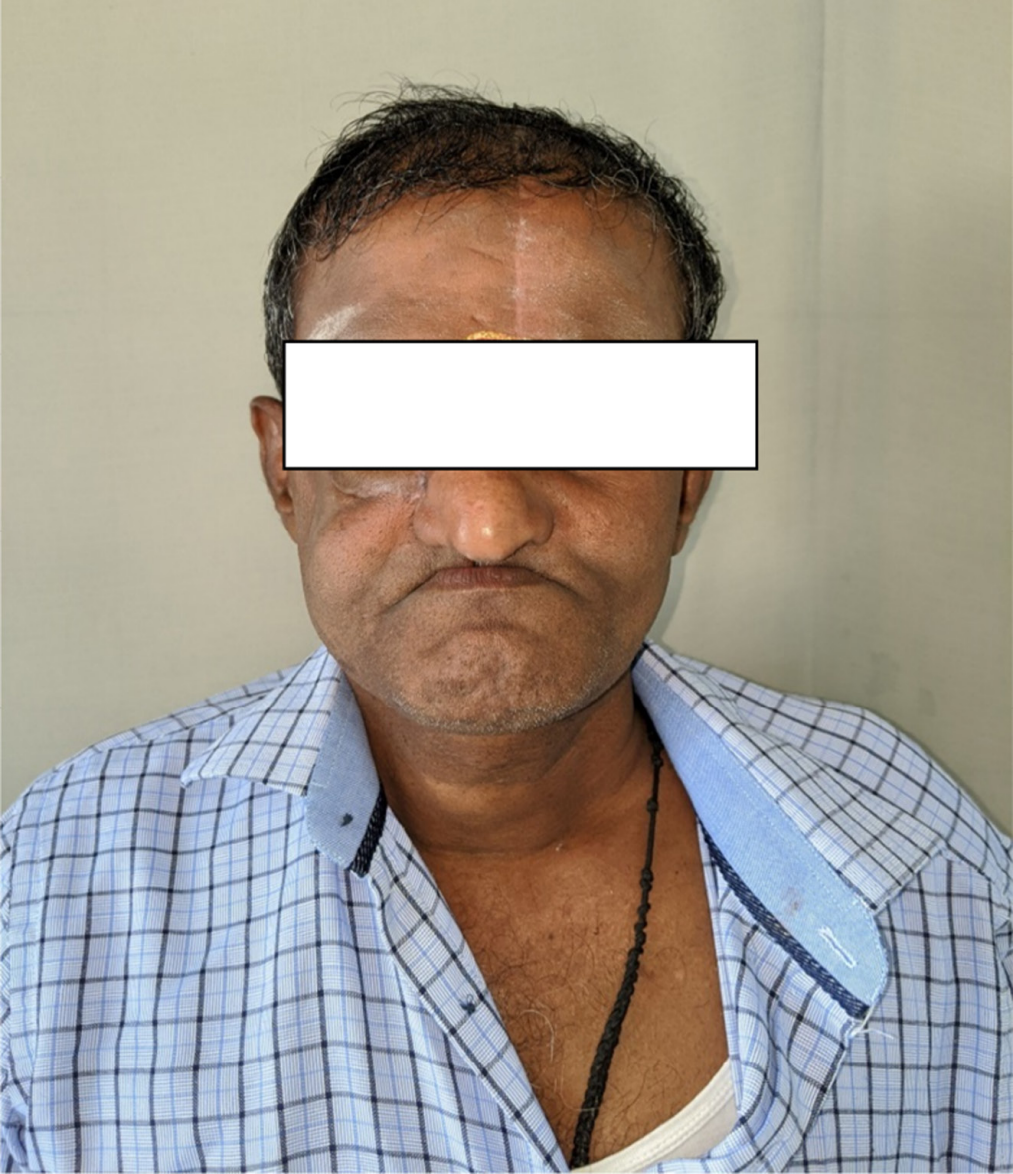
3‐month post‐operative view of the patient.

Correlating the history, and clinical, radiographic, and histopathological findings depicting the morphological presentation of the fungal hyphae, a definitive diagnosis of fungal osteomyelitis was made, suggesting mucormycosis. The patient was managed by total maxillectomy on the right side with zygomatic extension and reconstruction with temporalis flap and split‐thickness skin graft and on the left side, subtotal maxillectomy. A periodic review was conducted which showed successful recovery posttreatment (Figure 10).

## 3 DISCUSSION

The present article highlights a case of COVID‐associated mucormycosis in a known diabetic patient who had a previous history of fever. However, it is to be noted that the patient did not check himself for COVID‐19 during the episode of fever and it is quite likely that it could be due to coronavirus infection which was prevailing all over the country. This could have precipitated the mucormycosis infection in the patient given his already immunocompromised diabetic state.

The standard recommendation to establish the diagnosis of CAM is by confirming COVID‐19 using RT‐PCR test along with clinical, radiographical, microbiological, and histopathological evidence of mucormycosis.^8^ In the present case, the patient reported to our institution after his episode of fever, for which he did not undergo any test. This is a common scenario in middle‐ and low‐income countries like India where diagnostic tests like RT‐PCR are often neglected and with the lack of availability of serum antigenic biomarkers, the diagnosis of CAM becomes challenging. However, it was decided to do the SARS‐CoV‐2 antibody test for the patient in the institution to confirm the diagnosis as the fever was almost a month old. This would provide information if the patient has a prior history of COVID‐19 as antibodies are known to remain in the patient's blood for many months. The presence of IgM antibody in his results confirmed a previous history of coronavirus infection and that too, a recent one. In the oral cavity, toothache, mobility of teeth and jaw, and bone exposure with necrosis are the usual manifestations of mucormycosis which was evident in the present case. The radiographical and histopathological findings of the patient corroborated the same further affirming the diagnosis of CAM. The patient was treated following the standard management protocol for mucormycosis.^10^, ^11^ Although amphotericin B is the drug of choice as the first line of therapy, the reported patient was started on Tab. Posaconazole, as amphotericin B, could not be procured during that time due to the wanting of the drug in the wake of CAM in India.^8^ However, the patient was found to respond well to the drug administrated. A multicentric study done by Patel et al. revealed an increased incidence of toothache, loosening of teeth, and radiologic involvement of the jaw in CAM patients as compared to non‐CAM patients. In fact, one of the centers reported jaw involvement in one out of every five cases of CAM.^2^ This evidence is in perfect concurrence with the present report and highlights the gravity of the disease. Furthermore, uncontrolled diabetes was found to be the most common underlying condition in CAM which was also in line with the present case.^2^

In addition, COVID‐19 is associated with an overexpression of inflammatory cytokines (cytokine storm) and impaired cellular immunity due to reduced T lymphocytes, and decreased CD4^+^ and CD8^+^ helper cells which makes patients with COVID‐19, an easy target for secondary infections.^1^ Several published reports have suggested the correlation between pre‐existing or steroid‐induced diabetes mellitus in COVID‐19 patients with increased chances of acquiring mucormycosis.^1^, ^5^, ^9^ In the present case too, the patient had a history of uncontrolled diabetes, was diagnosed with COVID‐19, and later developed oral mucormycosis.

The association between diabetes, mucormycosis, and COVID‐19 could be explained as follows: uncontrolled hyperglycemia and precipitation of ketoacidosis often seen in patients under steroids, leads to a fall in pH due to acidosis providing a fertile environment for the germination of the fungal spores. Moreover, the use of steroids also reduces the first and second line of defense mechanism leading to impairment in the chemotactic and phagocytic activity of the bronchoalveolar macrophages, making a diabetic patient particularly vulnerable to mucormycosis.^9^ Furthermore, hyperglycemia leads to glycosylation of transferrin and ferritin, reducing the iron binding capacity and causing increase in free iron. Concomitant acidosis also increases free iron by the same mechanism impairing the ability of transferrin to chelate iron. In addition, increased cytokines (IL‐6) in patients with COVID‐19 escalates the free iron content in the blood by increasing the ferritin levels via increased synthesis and reduced iron transport. All these factors prove to be ideal resources for the growth of mucormycosis.^9^ In addition, studies have also suggested that inadvertent use of corticosteroids (long‐ and short‐term) could predispose to CAM, especially in patients with altered immunity.^6^ Therefore, it is quite evident that the combination of diabetes, steroids, and mucormycosis forms an unholy trinity in patients with COVID‐19.^6^

In addition, critically ill patients requiring extensive hospitalization in ICU with or without mechanical ventilators are ideal to colonize opportunistic microorganisms.^1^ In a study conducted in one of the hospitals in Mumbai, India, it was seen that 30% of patients developed symptoms of mucormycosis within 12 days of hospitalization, the earliest being 5 days. The prevalence of CAM cases in ICU, as reported by Patel et al., was 1.6% and was associated with a greater mortality rate.^2^ The probable cause of this issue could be the lack of inadequate sterilization of the ICU, bedside contamination, unhygienic delivery of oxygen to the COVID patients, and use of contaminated oxygen masks among patients due to the large influx of COVID cases during the time. Zinc is commonly prescribed to COVID‐19 patients to boost their immunity. However, zinc is reported to be associated with CAM as it provides a favorable environment for the growth of the fungus while zinc chelators (antidote) such as clioquinol or phenanthroline neutralize the growth of the fungus.^6^

While the patient had an uneventful recovery following the treatment, confirmation of COVID‐19 diagnosis without RT‐PCR remains a major limitation. However, under the challenging circumstances of the second wave of the COVID‐19 pandemic and with patients reporting when the episode of fever is over, antibody testing could prove beneficial for confirming a prior infection so that an appropriate diagnosis can be made and effective treatment can be provided to the patient.

## 4 CONCLUSION

Coronavirus‐associated mucormycosis (CAM) had reached an endemic status during the second wave of the COVID‐19 pandemic with increasing mortality rates. Therefore, it is imperative that clinicians and dental professionals, in particular, should be aware of the seriousness of the disease, its risk factors, and clinical presentations. Uncontrolled diabetes and overzealous use of steroids in the management of COVID‐19 are the triggering factors of mucormycosis and should be closely monitored. Early diagnosis, immediate management with anti‐fungal drugs such as amphotericin B, posaconazole, or isavuconazole, and surgical debridement, if necessary, should be performed for patients with CAM. However, overtreatment with amphotericin B should be avoided as it can lead to serious complications such as nephrotoxicity.

## AUTHOR CONTRIBUTIONS

**Protyusha GB:** Conceptualization; investigation; writing – original draft. **Kavitha B:** Investigation; writing – review and editing. **B Sivapathasundharam:** Writing – review and editing. **P Manodh:** Investigation; writing – review and editing. **A. Thirumal Raj:** Writing – review and editing. **Snehashish Ghosh:** Conceptualization; writing – original draft; writing – review and editing. **Safal Dhungel:** Writing – review and editing.

## CONFLICT OF INTEREST STATEMENT

The authors declare no conflict of interest.

## ETHICS STATEMENT

Ethical approval was not required from the institution, in accordance with our country's law, as this was a case report.

## CONSENT

Written informed consent was obtained from the patient to publish this case report in accordance with the journal's patient consent policy.

## Data Availability

The data that support the findings of this article are available from the corresponding author upon reasonable request.
